# Case Report: A patient with lynch syndrome with vaginal endometriosis-associated malignancy and synchronous colonic tubulovillous adenoma

**DOI:** 10.3389/fmed.2025.1672641

**Published:** 2025-10-08

**Authors:** Ting Kuang, Xiaoping Liu, Qi Li, Meiyuan Huang, Ziqian Tang, Xidie Li, Jinjin Wang, Huan Chen

**Affiliations:** ^1^Department of Obstetrics and Gynecology, Zhuzhou Central Hospital, Zhuzhou, Hunan, China; ^2^Department of Pathology, Zhuzhou Central Hospital, Zhuzhou, Hunan, China

**Keywords:** lynch syndrome, extra-ovarian endometriosis-associated cancer, vagina, tubulovillous adenoma, case report

## Abstract

Endometriosis is a common gynecological condition. However, endometriosis-associated malignancies occur in up to 1% of women with endometriosis. Most cases of endometriosis-associated malignancy occur in the ovary, whereas 20% of cases occur at extragonadal sites. Herein, we report the case of a patient with an incidental finding of vaginal endometriosis-associated malignancy who was later diagnosed with Lynch syndrome due to MSH6 deletion with loss of protein expression and was subsequently found to have a high-grade colonic tubulovillous adenoma. The patient was a 50-year-old (Para 2 + 2) woman without any previous history suggestive of adenomyosis or endometriosis, who was examined at a local hospital and was found to have swelling in the posterior vaginal fornix. Colposcopy was performed, and the mass was biopsied, revealing endometrial adenocarcinoma. She was then transferred to our hospital, where, after a series of assessments, she underwent surgery (including total hysterectomy, double adnexectomy, partial vaginal hysterectomy, and lymph node dissection). A postoperative pathological examination indicated a diagnosis of vaginal endometriosis-associated malignancy. A paclitaxel/carboplatin (TC) regimen (paclitaxel 175 mg/m^2^ + carboplatin AUC 5) was initiated 9 days postoperatively. Loss of MSH6 protein expression in the mass was observed using postoperative immunohistochemistry. Genetic sequencing revealed pathogenic MSH6 variants, including p. F1104Lfs*11 (c.3312delT) and c.3556 + 1G > A, indicating germline mutations. These findings suggest the presence of Lynch syndrome. Before the second postoperative chemotherapy cycle, the patient underwent a colonoscopy, and a mass measuring approximately 6 cm in diameter was identified in the right half of the transverse colon. One month after the second cycle of chemotherapy, the patient underwent laparoscopic radical right hemicolectomy. Histopathological examination revealed a tubulovillous adenoma with high-grade intraepithelial neoplasia of partial glands. One month after the second surgery, the patient was referred to our department, completed four cycles (a total of six cycles) of combination chemotherapy (carboplatin and paclitaxel), and was recurrence-free at the last follow-up (July 2025). Lynch syndrome with both extra-ovarian endometriosis-associated cancer and intestinal lesions is rare. We report a case of incidentally identified vaginal endometriosis-associated malignancy in a patient with Lynch syndrome due to MSH6 protein deficiency and MSH6 germline mutations and the discovery of a high-grade tubular choriocapillaris adenoma of the colon. This case highlights the critical importance of MMR protein testing in the screening for Lynch syndrome in patients with extra-ovarian endometriosis-associated cancer and preoperative colonoscopy. Although there are no established guidelines for the treatment of extra-ovarian endometriosis-associated cancer, its management is currently based on protocols for the treatment of primary ovarian cancer.

## Introduction

Endometriosis-associated malignancy (EAM) occurs in up to 1% of women with endometriosis. Most cases of EAM occur in the ovary, whereas approximately 20% occur in extragonadal sites, including the intestine, the rectovaginal septum, the abdominal wall, and the pleura. The diagnostic criteria for EAM proposed by Sampson (1) in 1925 include the following: (1) coexistence of cancerous and endometriotic tissue in the same lesion; (2) histological correlation between the two, with endometrial mesenchymal-like tissue surrounding characteristic endometrial glands or old hemorrhages; and (3) exclusion of other primary tumors or cancerous tissue occurring in endometriotic foci rather than metastasis by infiltration from other sites. In 1953, Scott added another diagnostic criterion (2): morphological evidence of the transformation of endopathy to malignancy or attachment of benign endometriotic tissue to malignant tumor tissue. Individuals with Lynch syndrome have genetic mutations that increase the risk of developing bowel, ovarian, and endometrial cancers (3). However, studies have also found that women with mutations in mismatch repair (MMR) genes have an increased risk of extra-uterine or extra-ovarian malignancies such as cervical cancer (4). A previous study reported a case of Lynch syndrome-related clear cell carcinoma of the cervix (5). This study reports a rare case of Lynch syndrome (LS) with EAM in a virginal patient with concurrent colonic lesions.

## Case description

**Figure 1:**
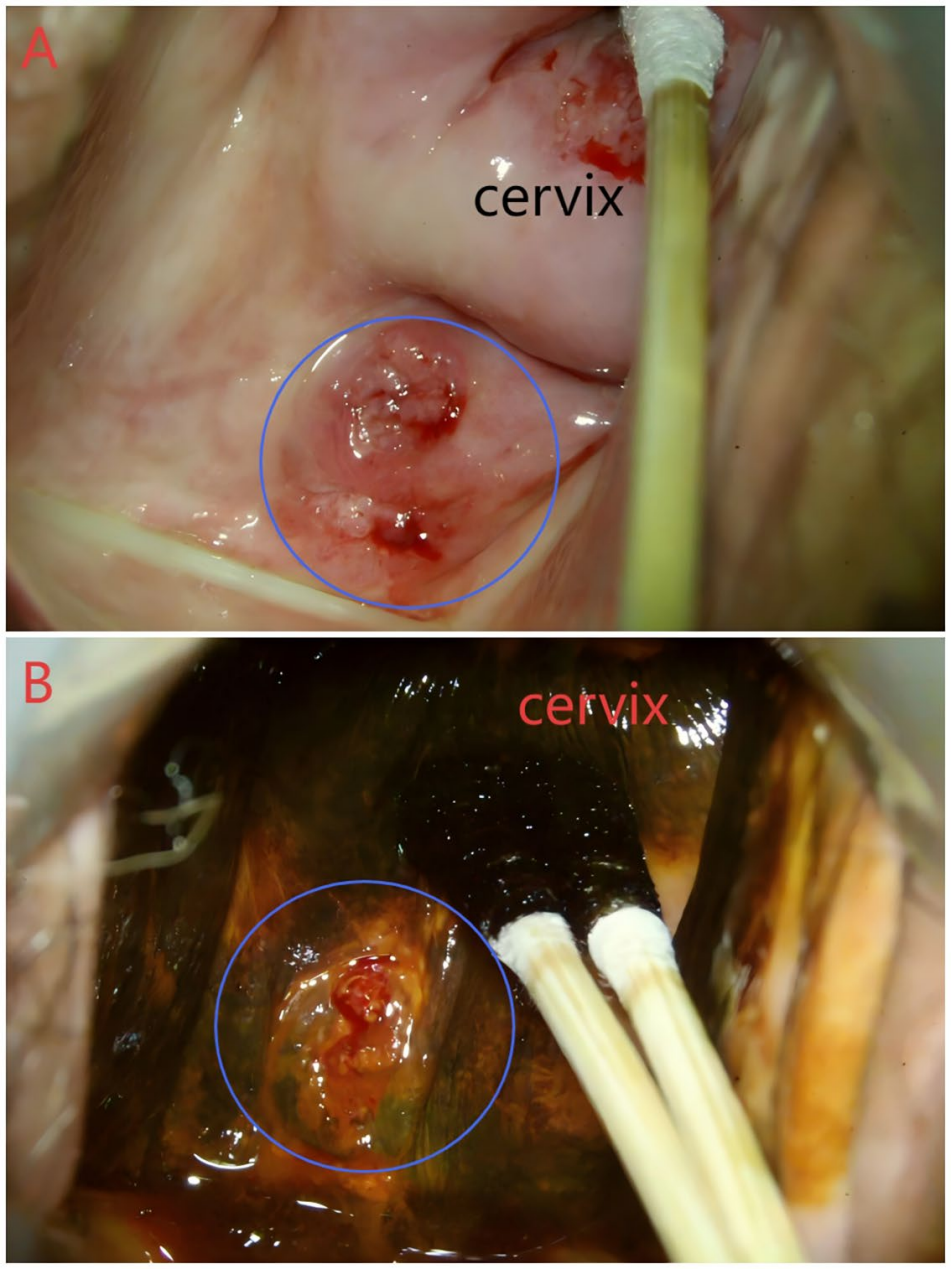
Colposcopy image of the patient. **(A)** Colposcopy image of the patient. A 1 × 1.5 cm^2^ cauliflower-like bulge was noted in the posterior fornix of her vagina. **(B)** Colposcopy image with iodine staining of the patient. The area within the blue circle indicates the bulge.

The patient was a 50-year-old woman (para 2 + 2) who was 7 months postmenopausal and had no history of abnormal vaginal bleeding. She had no previous history of dysmenorrhea, adenomyosis, endometriosis, hormone treatment, surgery, radiotherapy, chemotherapy, alcohol use, smoking habits, or a family history of tumors. In early November 2023, during cervical and breast cancer screening at a local hospital, a swelling was detected in the posterior vaginal fornix. Colposcopic evaluation revealed a Type 3 transformation zone in the cervix. Although the appearance of the cervix was normal, a 1 × 1.5 cm^2^ cauliflower-like swelling was noted in the posterior fornix of the vagina (Figures 1A,B). A biopsy of the mass and endocervical curettage (ECC) were performed, revealing benign glandular tissue. Further pathological evaluation of the mass revealed active hyperplasia of the glandular epithelium, mild-to-severe atypical hyperplasia, and focal carcinogenesis. The immunophenotype profile of the cancer cells (P16 focal+, ER 90%++ ~ +++, PR 10%++, C-erB-2+, PAX8+, P53 mutation-type expression, Ki-67 50%, and PD-L1 CPS10) supported the diagnosis of endometrial adenocarcinoma (Table 1).

**Table 1:** Timeline table.

Date	Event	Significance
November 2023	Swelling was discovered in the posterior vaginal fornix during cervical and breast cancer screening	Biopsy confirmed endometrial adenocarcinoma.
20 December 2023	Patient was transferred to our hospital	
22 December 2023	Surgery	Total hysterectomy, bilateral salpingo-oophorectomy, lymphadenectomy, and partial vaginectomy
27 December 2023	Pathology: Malignant transformation of vaginal endometriosis	IHC showed MSH6 loss.
3 January 2024	First postoperative chemotherapy cycle.	Paclitaxel 175 mg/m^2^ + Carboplatin AUC 5
17 January 2024	Genetic testing confirmed germline *MSH6* mutation (p. F1104Lfs*11, c.3556 + 1G > A)	Diagnosed with Lynch syndrome
23 January 2024	Colonoscopy revealed a 6-cm mass in the transverse colon	
26 January 2024	Second chemotherapy administered	Paclitaxel 175 mg/m^2^ + Carboplatin AUC 5
28 February 2024	Laparoscopic radical right hemicolectomy	The mass was pink with a 4 cm diameter
3 March 2024	Pathology: tubulovillous adenoma with high-grade intraepithelial neoplasia.	
April–June 2024	Completed four cycles of chemotherapy.	Paclitaxel 175 mg/m^2^ + Carboplatin AUC 5
July 2025	Last follow-up: no recurrence or complications.	

## Diagnostic assessment

On 21 December 2023, the patient was transferred to our hospital for further management. The biopsy of the patient’s vaginal posterior fornix mass indicated a malignant tumor; therefore, we first considered the possibility of a vaginal malignant tumor. The patient’s pathological findings revealed an endometrioid adenocarcinoma, whereas 85–95% of primary vaginal cancers are squamous cell carcinomas; therefore, we did not consider a primary vaginal cancer and considered the possibility of a metastatic tumor. Physical examination of the patient upon admission revealed no abnormalities. Except for swelling in the posterior vaginal wall, no abnormalities were observed during the gynecologic and digital rectal examinations. She was then admitted and underwent a series of tests. A thin-layer liquid-based cytological test (TCT) and cervical HPV testing were both negative. Tumor markers were also within normal limits: AFP 2.34 ng/mL, CEA 0.6 ng/mL, CA-125 9.98 U/mL, and HE4 39.76 pmol/L. A 21 × 19-mm cystic lesion was observed in the left ovary on B-ultrasonography, the right ovary was atrophic, and multiple hypoechoic nodules without intranodular blood flow were observed in the myometrium, with the largest measuring 18 × 12 mm. The uterine cavity was empty, and the thickness of the endometrium was 3–4 mm without a blood flow signal. Computed tomography (CT) of the chest, abdomen, and pelvis only revealed a 16 × 15 mm benign-looking cystic lesion in the left adnexa and a suspicious 14 × 12 mm intramural uterine myoma; no other masses or lymph node enlargement were found. Similar findings were observed on pelvic MRI; however, MRI also revealed a 9 × 7 mm lesion with slightly prolonged T1 and T2 signals, diffusion restriction, and contrast enhancement in the upper posterior vaginal wall. The endometrial thickness was normal without abnormal enhancement.

**Figure 2:**
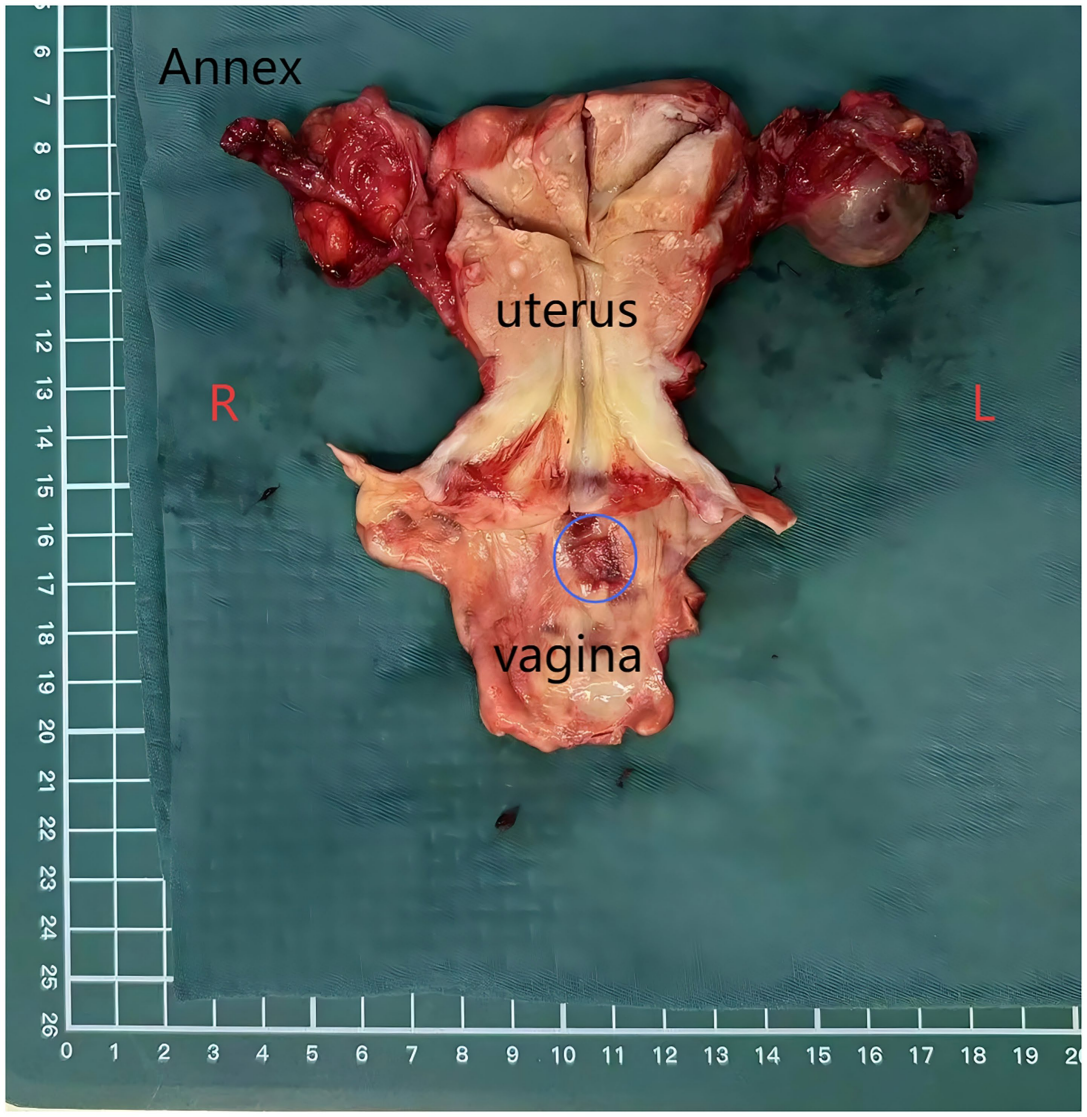
Specimen of the first operation. The lesion is located in the posterior fornix of her vagina (blue circle).

**Figure 3:**
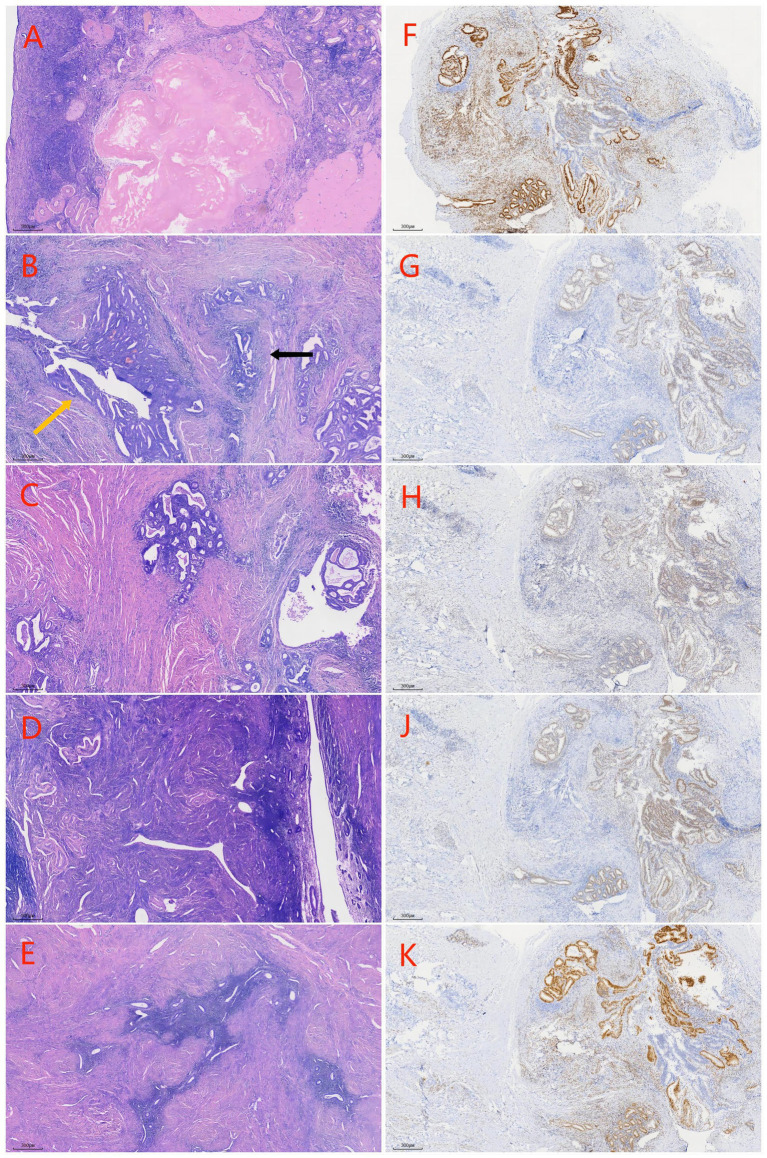
Pathological morphology features and immunohistochemistry results of the patient. **(A)** Ovary; **(B)** carcinomatous foci and ectopic endometrium (yellow arrow: carcinomatous foci, black arrow: ectopic endometrium) **(C)** ectopic endometrium of vagina (black arrow: ectopic endometrium); **(D)** atrophic endometrium (blue arrrow); **(E)** adenomyosis (orange arrow); **(F–K)** Immunohistochemistry staining pictures of the patient (ER, PR, MLH1, MSH2 and PMS2 were positive).

**Figure 4:**
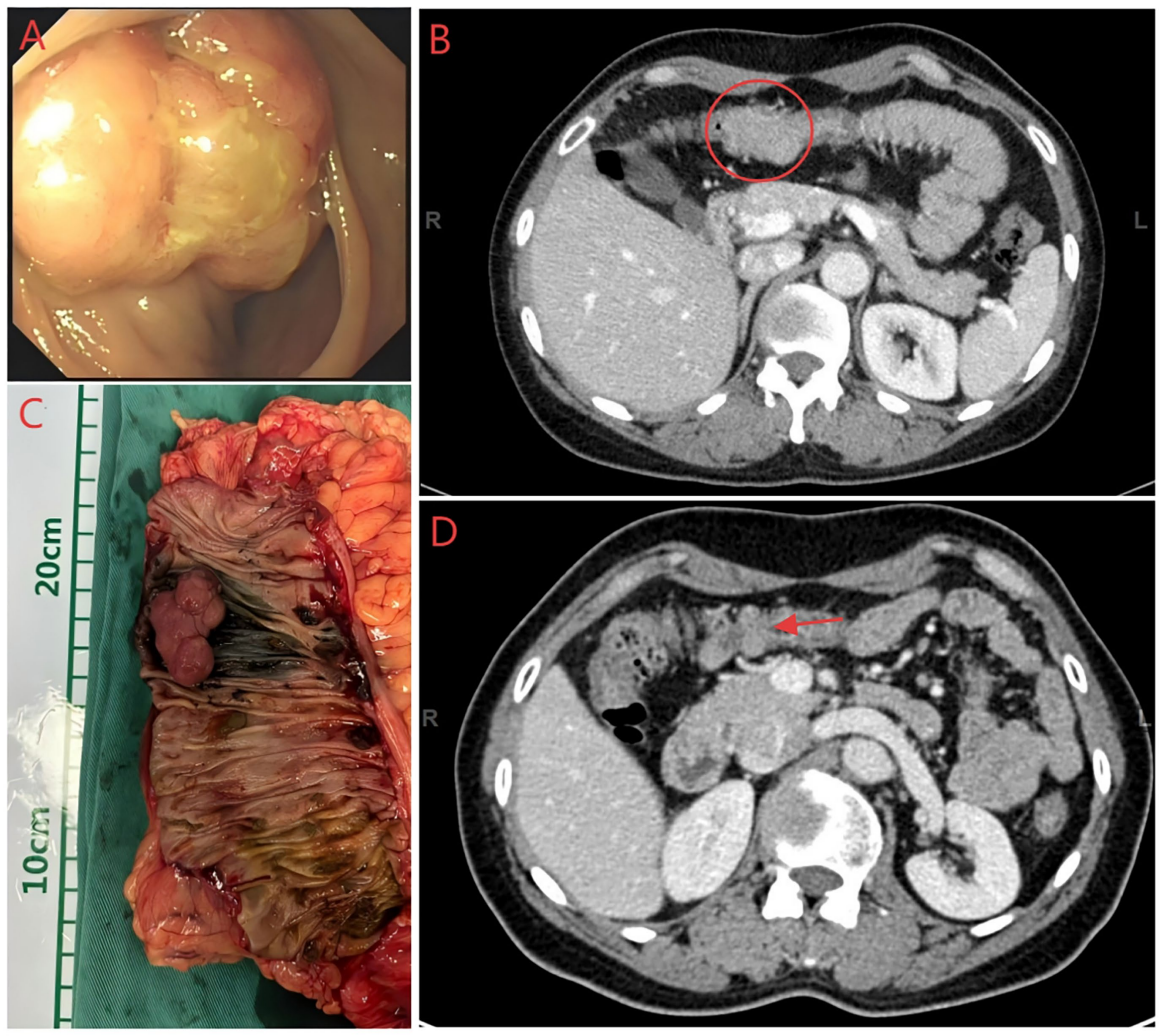
Colonoscopy, CT and colon specimen image of the patient. **(A)** Colonoscopy image of the patient. **(B)** Abdominal CT images before the second surgery, the mass is located within the red circle. **(C)** Intestine specimen after radical right hemicolectomy (A mass was pink with a 4 cm diameter). **(D)** Abdominal CT images before the first surgery, the area indicated by the red arrow is the suspected mass.

Surgical excision of the mass is considered the best treatment option. On 25 December 2023, the patient underwent surgery; to ensure a clean margin and complete tumor resection, the surgical treatment involved hysterectomy, bilateral adnexectomy, excision of the upper part of the vagina (Figure 2), and lymph node dissection. The surgery and postoperative courses were uneventful. On 30 December 2023, the postoperative pathological result suggested a moderately differentiated endometrial carcinoma of the vaginal fornix, and endometriotic components were observed around the tumor. The lesion involved the full thickness of the vaginal wall, with a close but clean deep margin. No malignant features were observed in the severed end of the vaginal wall, and no tumor plugs were seen, including uterine adenomyosis, atrophic endometrium, chronic inflammation of the bilateral fallopian tubes with left parovarian cyst and simple cysts of the left ovary, and no lymph node metastases (Figures 3A–E). Based on the pathological findings, the patient was ultimately diagnosed with EAM and staged as FIGO Stage IA (with reference to vaginal carcinoma). Immunohistochemistry revealed the following: MLH1(+), MSH2(+), MSH6(−), PMS2(+), ER(+), PR(+), P53(focal+), P16/mtsl(partial+), Vimentin(partial+), CEA(−), and Ki-67 (30%) (Figures 3F–K). Due to a total loss of expression of the MSH6 protein, the patient underwent genetic sequencing. On 3 January 2024, we initiated a paclitaxel/carboplatin (TC) regimen (paclitaxel 175 mg/m2 + carboplatin AUC 5) as postoperative chemotherapy. The first chemotherapy cycle was uneventful. On 17 January 2024, the genetic sequencing results showed that MSH6 p. F1104Lfs*11 (c.3312delT) and c.3556 + 1G > A were positive, indicating the presence of germline mutations. These findings strongly suggested a diagnosis of LS. On 20 January 2024, the patient was readmitted for the second cycle of chemotherapy. On 23 January 2024, she underwent a colonoscopy before the second postoperative chemotherapy treatment to rule out intestinal lesions. A mass of approximately 6 cm in diameter was identified in the proximal half of the patient’s transverse colon, and samples were obtained for biopsy (Figure 4A). On 26 January 2024, the patient underwent the second cycle of postoperative chemotherapy. Pathological examination of the biopsied colonic mass revealed a tubular adenoma, with focal glands and high-grade hyperplasia. One month later, the patient was admitted to the Department of General Surgery for treatment. A repeat abdominal CT scan showed a mass measuring 4 cm in diameter in the right half of the patient’s transverse colon (Figure 4B). The two CT images (Figure 4C) were compared, and a senior radiologist was consulted. On 28 February 2024, she underwent laparoscopic radical right hemicolectomy (Figure 4D). Histopathological evaluation of the mass revealed a tubulovillous adenoma with high-grade intraepithelial neoplasia of partial glands. The final staging was TisN0M0. The patient had an uneventful postoperative recovery.

From April to June 2024, the patient was closely followed up on at our department. The treatment plan remained unchanged, and the patient completed four cycles of six cycles of combination chemotherapy. The treatment was well tolerated, with only CTCAE grade 2 alopecia and no other severe adverse events. Thereafter, the patient was followed up with regular quarterly visits; her last follow-up visit was in June 2025. Surveillance, including CT scans and an assay of tumor markers (CA-125), revealed normal findings.

## Discussion

In line with the criteria proposed by Sampson (1) in 1925 and the addition made by Scott in 1953 (2), the patient was diagnosed with EAM. Endometriotic lesions have an approximately 1% risk of malignant transformation. This malignancy is mainly of glandular epithelial origin, is located in the ovary, and is referred to as endometriosis-associated ovarian cancer (EAOC). Malignancies associated with endometriosis at other sites, such as the vaginorectal septum, the abdominal wall, or the perineal incision, are less frequent and are often called extra-ovarian endometriosis-associated cancer (EOEAC). Endometriosis is a high-risk factor for endometrioid adenocarcinoma and clear-cell carcinoma of the ovary (6). Combined with postoperative pathology and medical history, our patient was diagnosed with EOEAC. Overall, the treatment of EOAC is currently based on the protocol for the management of primary ovarian cancer. Therefore, the treatment strategies for primary ovarian cancer and EAOC of the same pathological type are comparable (7). Typically, these therapies include surgery, chemotherapy, radiation therapy, targeted therapy, and hormonal therapy. Although there are currently no established therapeutic interventions for EAOC mutations, immunotherapy has been shown to be effective. Some studies have found that the following mechanisms are involved in the development of EAOC: abnormal expression of related genes, genetic regulation of miRNAs, oxidative stress, abnormal gene methylation, imbalance in hormonal regulation, imbalance of immune regulation, and inflammation (8). These studies provide potential targets for future EAOC treatments. However, there is no clear reference for EOEAC treatment. Therefore, the optimal management strategy for EOEAC remains poorly established due to limited experience.

The MMR system is important for maintaining fidelity after replication (9). The MMR system includes the MutS (MSH2, MSH3, MSH6, etc.) and MutL (MLH1, MLH3, PMS1, and PMS2) families. Among these, MLH1, MSH2, MSH6, and PMS2 are the dominant MMR proteins. A lack of expression of ≥1 of these four major MMR proteins leads to defective mismatch repair (dMMR). A heterozygous inactivating mutation in any of the four MMR genes (MLH1, MSH2, MSH6, and PMS2) results in LS, which causes hereditary nonpolyposis colorectal cancer (10). In our patient, immunohistochemistry revealed deletion of MSH6 expression, and subsequent genetic sequencing revealed germline mutations in MSH6, both of which suggested that she had LS. However, we were puzzled as to why the preoperative enhanced CT did not reveal the mass in the transverse colon. Certain studies have shown that a specialized CT scan called colonography has a 95% sensitivity comparable to colonoscopy; however, it requires bowel preparation, which some patients may find difficult to tolerate. An alternative method is CT colonography with oral contrast fecal tagging; however, the difficulty in identifying polyps without bowel preparation lowers its sensitivity to 72% (11). The patient was not re-evaluated using these special methods. Meanwhile, patients do not need to undergo bowel preparation when undergoing gynecological CT or MRI examinations, which may also be one of the reasons for the absence of positive results on the first CT scan. Therefore, preoperative enhanced CT did not reveal the mass because of a lack of experience on the part of the radiologist, inadequate bowel preparation, and interference from intestinal gas. However, immunohistochemistry of MMR protein was not performed on the patient’s preoperative biopsy specimen; therefore, the expression status of MMR-related genes could not be determined preoperatively. Another important reason is the lack of thoroughness in the preoperative physical examination; our patient had no family history of any tumors; hence, a colonoscopy was not recommended preoperatively, which led to a delay in diagnosis. Therefore, even if no abnormalities are identified during preoperative imaging examinations, diagnostic colonoscopy is necessary for patients with known LS or for those at high risk.

LS is one of the most common causes of inherited cancers resulting from hereditary defects in MMR genes (12). Gynecological malignancies are usually the precursor tumors of LS in women, and endometrial cancer (EC) is the most closely associated cancer. Approximately 70–90% of LS cases are caused by deletions in the MLH1 and MSH2 genes (13). Genetic mutations prevent MMR during cellular DNA synthesis, leading to highly recombinant DNA sequence instability in the genome, known as microsatellite instability (MSI), which is an important cause of tumorigenesis and progression (14). MMR mutations can occur in all cells. This might be the cause of the malignant transformation of the ectopic endometrium in such patients. A previous study characterized 666 endometrial carcinoma (EC) cases using immunohistochemistry (IHC), microsatellite instability (MSI) testing, and mut-L homolog 1 (MLH1) methylation. MSI testing revealed that 27.3% of the cases were MSI-high (*n* = 182), whereas MMR IHC identified 25.1% of the cases as MMR-deficient (*n* = 167), with 3.8% of the cases (*n* = 25) showing inconsistent results. An analysis of IHC staining revealed inconsistent findings in 18 cases, showing subclonal loss of MLH1/PMS2 (*n* = 10) and varied MMR IHC (MSH6, *n* = 7; MLH1/PMS2, *n* = 1). In three out of six cases, LS linked to MSH6 had a heterogeneous expression (15). Consequently, the incidence of LS caused by MSH6 is not high. Other studies have analyzed the cancer risk associated with germline mutations in the MSH6 gene in LS. For carriers of MSH6 mutations, the estimated cumulative risk of colorectal cancer by the age of 70 years was 12% (95% CI, 8–22%), that of endometrial cancer was 16% (95% CI, 8–2%), and that of ovarian cancer was 1% (95% CI, 0–3%). By the age of 40, the estimated cumulative risk of endometrial cancer in mutation carriers does not exceed 2% (95% CI, 0–7%), whereas the cumulative risk of ovarian cancer does not exceed 1% (95% CI, 0–3%) (16). Ovarian endometriosis can lead to the development of clear cell and endometrioid carcinomas (17); 27% of endometriosis-associated ovarian cancers are ovarian endometrioid carcinomas (18), whereas approximately 40% of LS-associated ovarian cancers are ovarian endometrioid carcinomas (19). The patient in our study had no reported history of endometriosis or adenomyosis; however, it is unclear whether the endometrium relocated to the vagina during implantation at delivery or implantation through the rectovaginal septum.

This report details a rare dual-pathology presentation of LS and highlights its critical screening implications in this population. To the best of our knowledge, there was only one case report of deep infiltrating endometriosis with malignant invasion of the cervical and rectal walls in a patient with LS (20). The patient had significant gastrointestinal symptoms, including diarrhea, and an MRI showed a mass possibly invading the anterior wall of the rectum. Therefore, the patient underwent a timely colonoscopy. On the other hand, the patient in our study exhibited no gastrointestinal symptoms, and MRI results showed that the lesion in the posterior vaginal wall had not invaded the rectum. This was also one of the reasons why the patient was not scheduled for a colonoscopy.

Although our patient was asymptomatic, some studies have addressed abnormal bleeding as a possible early clinical manifestation (21). Currently, screening methods for LS-related gynecological malignancies mainly consist of two parts: clinical and molecular biological screening. Clinical screening involves collecting patients’ personal and family medical histories to conduct a selective initial screening for individuals suspected of having LS. However, there are multiple molecular screening methods available for the screening of LS, including (1) IHC: It detects whether the expression of the four proteins (MLH1, MSH2, MSH6, and PMS2) encoded by the MMR gene is normal. Studies have shown that the sensitivity and specificity of IHC for detecting LS are 91 and 83%, respectively, which are superior to those of MSI testing (22), making it the preferred screening method for LS-related gynecological malignancies. (2) MLH1 promoter demethylation detection. (3) MSI testing: It shows a high degree of consistency with IHC. In a study by Carnevali et al. (23), the consistency between the two methods was 94.5%. (4) Germline MMR Gene Testing: Based on the results of initial IHC screening or MSI testing, germline MMR gene mutation testing performed on patients’ blood samples is currently the gold standard for diagnosing LS.

Some of this study’s limitations include its retrospective nature and the initially missed colonic lesions on imaging. First, the patient should undergo a colonoscopy before the first operation. Second, the CT films should have been interpreted by an experienced radiologist. Third, the gastrointestinal endoscopists may have been conservative. A biopsy of the pathological lesion revealed a high-grade intraepithelial neoplasia; however, the presence of deep submucosal infiltration and regional lymph node metastasis should have been further determined using techniques such as fine-tuning or ultrasonographic endoscopy. Furthermore, endoscopic resection is recommended if the lesion does not show deep submucosal infiltration or regional lymph node metastasis. For intramucosal cancers without lymph node metastasis and SM1 stage cancers with mild infiltration into the submucosa, endoscopic treatment is comparable to surgery (24) with fewer complications, shorter duration of hospital stay, and lower costs (25). These factors may have allowed the patient to avoid a second surgery.

For the treatment of such patients, the protocol for the management of EAOC is the only currently available guide. Our patient underwent surgery and received six cycles of chemotherapy. Because she was positive for both estrogen and progesterone receptors, hormonal therapies could have also been considered. Moreover, the role of the tumor microenvironment, estrogen signaling, oxidative stress, and inflammation in the pathogenesis of EAOC offers new therapeutic strategies, such as targeted therapies and immunotherapy (7).

In summary, based on our report of this rare case and available reports in the literature, MMR protein testing should be performed while screening for LS in patients with EAM (especially in non-ovarian sites). Patients with a confirmed diagnosis of LS or those at a high risk of LS must undergo a complete colonoscopy prior to gynecological oncological surgery. Additionally, in the absence of EOEAC-specific guidelines, the treatment strategy for primary ovarian cancer (surgery + combination chemotherapy) is a reasonable alternative.

### Patient perspective

The patient expressed profound anxiety upon diagnosis of vaginal malignancy, which was relieved after successful treatment. She emphasized the importance of clear communication about the multi-organ risks of LS and endorsed preoperative colonoscopy for similar patients.

## Data Availability

The original contributions presented in the study are included in the article/supplementary material, further inquiries can be directed to the corresponding author.
